# Outcomes of intramedullary nailing versus plate fixation for humeral shaft fractures: a retrospective cohort study

**DOI:** 10.1007/s00590-025-04181-z

**Published:** 2025-02-07

**Authors:** Jawad Derbas, Isam Moghamis, Osama Alzobi, Amgad Elshoeibi, Abdullah Murshid, Ghalib Ahmed

**Affiliations:** 1 Department of Orthopedic Surgery, Hamad General Hospital, Doha, Qatar; 2 College of Medicine, QU Health, Qatar University, Doha, Qatar

**Keywords:** Humerus shaft, Fracture, DCP, IMN, Functional outcome

## Abstract

**Background:**

Humeral shaft fractures account for 1–3% of all bone fractures. Conservative treatment often leads to complications such as non-union and shoulder stiffness. Surgical fixation with a dynamic compression plate (DCP) has been the gold standard treatment. Intramedullary nailing (IMN) has recently gained popularity due to its minimally invasive approach and reduced risk of radial nerve injury. This study aims to compare the outcomes of IMN and plate fixation for humeral shaft fractures.

**Methods:**

This retrospective study included patients with humeral shaft fractures treated with either IMN or DCP fixation at Hamad General Hospital between April 2015 and October 2018. Patient demographics, fracture characteristics, surgical outcomes, and complications were collected. Descriptive statistics were used to summarize patient information, and univariate analysis was conducted to compare both groups. A Cox proportional hazards model, adjusted for age, gender, and polytrauma status was applied to compare time to union between IMN and DCP groups.

**Results:**

Sixty five patients (25 IMN, 40 plate fixation) were included. Non-union rates were higher in the DCP group than in the IMN group (13% vs. 4%). Reoperation rates were also higher in the DCP group (20% vs. 4%). Postoperative neuropathy rates were 4% for IMN and 10% for DCP, with neuropathy resolution significantly higher in the IMN group (92% vs. 68%). Shoulder range of motion (ROM) and pain favored the DCP group, with 98% unaffected ROM in the plate group compared to 76% in the IMN group (*p* = 0.007), and a lower incidence of shoulder pain (28% vs. 98%, *p* < 0.001). Time to union was comparable between both groups, with an adjusted hazard ratio of 1.08 (95% CI 0.62–1.90; *p* = 0.776).

**Conclusion:**

IMN and plate fixation effectively achieved fracture union; however, plate fixation was associated with better shoulder function, reduced pain, and higher reoperation rates. IMN was linked to a lower risk of nerve injury but compromised shoulder ROM and resulted in more postoperative pain.

## Introduction

Humeral shaft fractures account for 1–3% of all bone fractures [1, 2]. These fractures can often be managed successfully with various casting techniques [3–5]. However, surgical intervention is typically indicated in specific cases, including open fractures, unstable fracture patterns, pathological fractures, and fractures associated with vascular injuries. It is also recommended for bilateral humerus fractures, polytrauma patients, and ipsilateral forearm fractures (floating elbow). Additionally, significant soft tissue injuries that prevent casting and failure of conservative treatment may necessitate surgical management [2].

Conservative management requires prolonged immobilization, which may not be suitable for many patients due to drawbacks such as shoulder stiffness and non-union rate ranging from 10 to 32%, which may be challenging to treat [3, 6–9]. As a result, there is a growing trend among orthopedic surgeons to opt for surgical treatment to avoid bracing complications and to facilitate earlier mobilization.

Open reduction and internal fixation (ORIF) using plates and screws has long been considered the gold standard for treating humeral shaft fractures [10]. However, this approach involves extensive soft tissue dissection, with a risk of radial nerve injury [11]. To mitigate these complications, advances in orthopedic techniques have made intramedullary nailing of the humerus an increasingly popular choice. This minimally invasive technique involves less soft tissue dissection and has a lower incidence of radial nerve palsy than ORIF [12–15].

This study aims to compare IMN and DCP for the management of humeral shaft fractures, focusing on operative outcomes, including complication rates, revision surgery, and time to union. We hypothesized that IMN may offer advantages in terms of reduced invasiveness, lower risk of infection, and potentially fewer nerve complications, while DCP may provide favorable outcomes for shoulder function.

## Materials and methods

### Study design and setting

This retrospective study was conducted following approval from the institutional review board of our local medical research center. The patient’s information was collected from their electronic medical records between April 1, 2015, and October 31, 2018. This included data on patient characteristics, fracture characteristics, types of fixations, complications, and postoperative follow-up information.

## Inclusion and exclusion criteria

All patients aged 16 years or older with acute humeral mid-shaft fractures who underwent treatment with either plate and screws or intramedullary nailing between April 1, 2015, and October 31, 2018, were included. Excluded from the study were patients whose primary mode of fixation was not a plate or nail, pediatric patients, patients with pathological fractures, and those with open fractures.

### Surgical technique

This study was conducted in a level 1 trauma center, and 12 different senior orthopedics trauma surgeons performed the surgeries with a minimum of five years of experience as attending physicians. The choice of the implant was primarily determined by the fracture configuration and the surgeon’s clinical judgment. The implants used for both fixation methods were standardized and widely accepted for treating humerus shaft fractures. A total of four proximal screws and four distal screws were used in the plating of the mid-shaft humerus. In the intramedullary nail fixation, none of the cases needed open reduction, and reduction was achieved by the closed technique, and the rotator cuff muscles were repaired following the insertion and fixation of the nail. The average time from the injury to the fixation of the fracture was approximately four days, based on the patient’s fitness to undergo surgery, especially in polytrauma cases.

### Data collection

Data collected for each patient included age, sex, mechanism of injury, fracture type, postoperative radiograph findings, and follow-up details such as functional outcomes, union rates, and reoperation rates. Additionally, infection rates, types of implants utilized in revision surgeries, time to union, presence of neuropathy, length of hospital stay, if the shoulder range of motion (ROM) was affected, and presence of shoulder pain were recorded. Neuropathy was categorized into two variables based on when it occurred (pre and postoperative) and whether it resolved. Nonunion was defined as a fracture that persists for at least nine months without signs of healing for three months on radiographs [16]. The sample size was not calculated for this study because all patients with humeral mid-shaft fractures during the study period and who met the inclusion criteria were included in this study, given the retrospective, cross-sectional nature of the study.

### Statistical analysis

All statistical analyses were conducted using Stata 17. The normality of continuous variables was assessed using histograms. Normally distributed variables were summarized as means and standard deviations, while skewed variables were summarized as medians and interquartile ranges (IQR). Student’s independent t-test was utilized to compare normally distributed variables, while the Wilcoxon Rank Sum test was used to compare skewed variables between the two groups. Categorical variables were reported as numbers and percentages.The Chi-square test or Fisher's exact test was used, as appropriate, to compare categorical variables between both groups. Cox proportional hazards model was performed to evaluate the time to union between the plate and IMN fixation groups, adjusting for age, gender, and polytrauma status. The hazard ratio (HR) for fixation type, along with its 95% confidence interval (95% CI), and exact p-value were reported.

### Ethical approval

This study was conducted in accordance with the Declaration of Helsinki (World Medical Association 2013) [17]. Data were de-identified and anonymized for analysis. Ethical approval was obtained from the local Institutional Review Board (MRC-01-21-024).

## Results

### Characteristics of participants

The baseline characteristics of the patients who underwent either IMN or plate fixation for humeral shaft fractures are summarized in Table 1. A total of 25 cases underwent IMN, and 40 were treated with plate fixation. The median age of the IMN cohort was 43.0 years (IQR 30.0–50.0) compared to the slightly younger median age of 34.5 years (IQR 28.0–39.0) in the plate fixation cohort. Gender distribution revealed a predominance of males in both groups, with 92% in the IMN cohort and 83% in the plate fixation cohort. There was a comparable distribution of diverse fracture patterns between both groups, including butterfly, severely comminuted, oblique, segmental, transverse, and short oblique fractures. There was a higher incidence of road traffic accidents (RTA) in the IMN cohort (60%) compared to the plate fixation cohort (70%). The prevalence of preoperative neuropathy was higher in the IMN cohort (96%) compared to the plate fixation cohort (78%). The total follow-up period and hospital stay were similar between both groups.

**Table 1:** Comparison of baseline characteristics between IMN and plate fixation of humeral shaft fractures

Variable	Sublevel	Overall	IMN	Plate	*p*-value
N		65	25	40	
Age median (IQR)		37.0 (29.0, 45.0)	43.0 (30.0–50.0)	34.5 (28.0–39.0)	0.021
Gender	Male	56 (86%)	23 (92%)	33 (83%)	0.28
	Female	9 (14%)	2 (8%)	7 (18%)	
Fracture configuration	Butterfly		4 (16%)	8 (20%)	0.27
	Severely Comminuted		4 (16%)	5 (13%)	
	Oblique		3 (12%)	14 (35%)	
	Segmental		2 (8%)	3 (8%)	
	Transverse		9 (36%)	6 (15%)	
	Short Oblique		3 (12%)	4 (10%)	
Mechanism of injury	RTA	43 (66%)	15 (60%)	28 (70%)	0.033
	Fall from height	9 (14%)	3 (12%)	6 (15%)	
	Pedestrian hit by car	4 (6%)	0 (0%)	4 (10%)	
	Fall from standing position	9 (14%)	7 (28%)	2 (5%)	
Polytrauma	No	35 (54%)	12 (48%)	23 (57%)	0.45
	Yes	30 (46%)	13 (52%)	17 (43%)	
Preoperative Neuropathy	No	61 (94%)	24 (96%)	31 (78%)	0.044
	Yes	4 (6%)	1 (4%)	9 (23%)	
Total follow-up period (months) median (IQR)		12.0 (10.0, 12.0)	12.0 (12.0–12.0)	12.0 (9.0–12.0)	0.50
Hospital Stay (days) median (IQR)		5.0 (4.0, 8.0)	6.0 (4.0–9.0)	5.0 (4.0–6.5)	0.39

### Surgical outcomes of IMN and plate fixation

Table 2 summarizes the surgical outcomes of patients who underwent either IMN or plate fixation for humeral shaft fractures. The non-union rates were slightly higher in the plate fixation group than in the IMN group (13% vs. 4%, respectively). Notably, there were no cases of malunion and infection in either group. Reoperation rates were higher in the plate fixation group compared to the IMN group (20% vs. 4%, respectively). The reasons for reoperation varied, including radial nerve exploration, ORIF revision, and plate removal. Postoperative neuropathy rates were 4% for IMN and 10% for plate fixation. Additionally, the resolution of neuropathy was significantly higher in the IMN group (92%) compared to the plate fixation group (68%). Shoulder ROM and pain outcomes differed between both groups, with DCP resulting in better shoulder ROM (98% unaffected in the DCP group vs 76% unaffected in the IMN group *p* = 0.007) and lower incidence of shoulder pain (28% vs. 98% *p* < 0.001) compared to plate fixation.

**Table 2:** Comparison of patient outcomes between IMN and plate fixation of humeral shaft fractures

Variable	Sublevel	Overall	IMN	Plate	*p*-value
Time to union mean (SD)		5.3 (2.3)	5.0 (1.3)	4.5 (0.9)	0.12
Non-union	No	59 (91%)	24 (96%)	35 (88%)	0.25
	Yes	6 (9%)	1 (4%)	5 (13%)	
Mal-Union	No	65 (100%)	25 (100%)	40 (100%)	
Infection	No	65 (100%)	25 (100%)	40 (100%)	
Reoperation	No	56 (86%)	24 (96%)	32 (80%)	0.42
	Radial nerve exploration	2 (3%)	0 (0%)	2 (5%)	
	ORIF revision	4 (6%)	1 (4%)	3 (8%)	
	Plate removal	1 (2%)	0 (0%)	1 (3%)	
	Tendon transfer	2 (3%)	0 (0%)	2 (5%)	
Postoperative neuropathy	No	59 (91%)	24 (96%)	36 (90%)	0.38
	Yes	6 (9%)	1 (4%)	4 (10%)	
Neuropathy resolved	No Neuropathy	50 (77%)	23 (92%)	27 (68%)	0.045
	Resolved	8 (12%)	2 (8%)	6 (15%)	
	Unresolved	7 (11%)	0 (0%)	7 (18%)	
Shoulder ROM	Unaffected	58 (89%)	19 (76%)	39 (98%)	0.007
	Affected	7 (11%)	6 (24%)	1 (3%)	
Shoulder pain	No	46 (71%)	7 (28%)	39 (98%)	< 0.001
	Yes	19 (29%)	18 (72%)	1 (3%)	

### Comparison of time to union between IMN and plate fixation using cox proportional hazards model

**Fig. 1:**
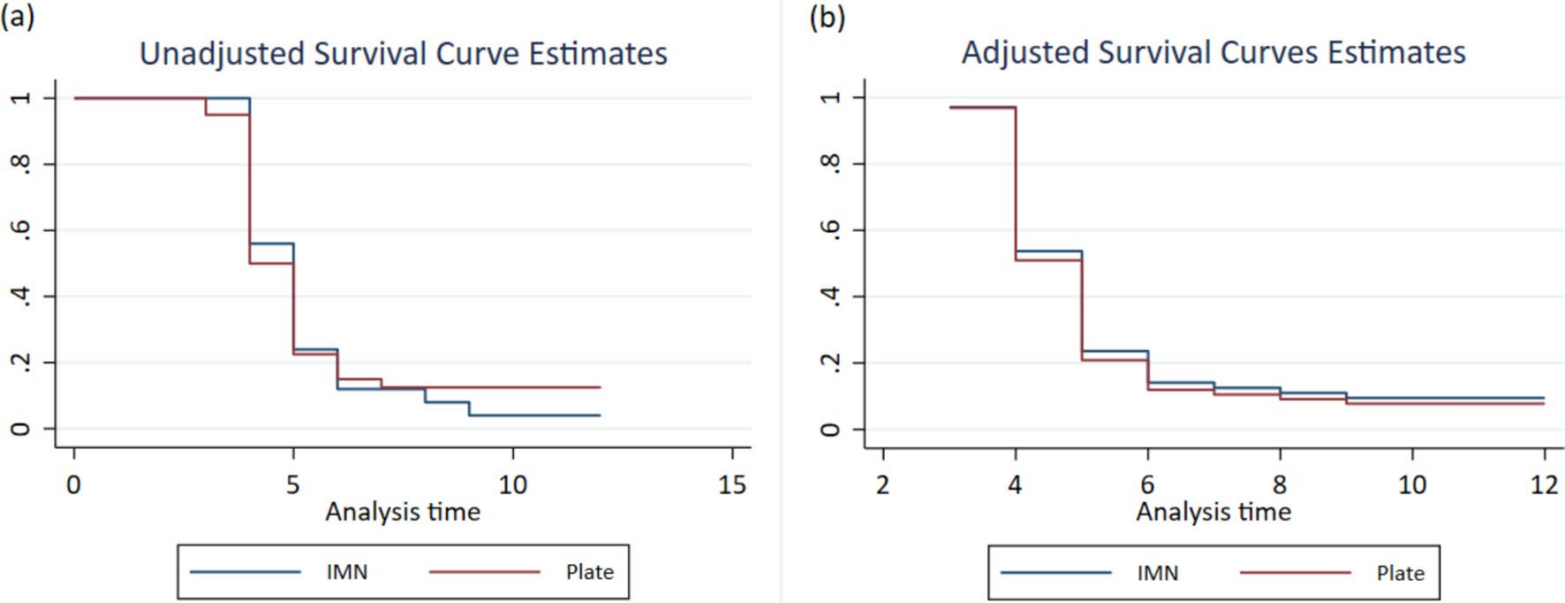
Survival curve for time to union by type of fixation: unadjusted (Panel A), adjusted using Cox model for age, gender, and polytrauma status

The association between fixation type and time to union for humeral shaft fractures was evaluated using a Cox proportional hazards model, with results presented in Table 3. In the unadjusted analysis, the hazard ratio for plate fixation compared to IMN fixation was 0.96 (95% CI 0.57–1.62; *p* = 0.893), indicating no significant difference between fixation types regarding time to union. The Kaplan–Meier survival curve (Fig. 1a) supports this, as the trajectories for plate and IMN groups show similar union times without notable divergence. After adjusting for age, gender, and polytrauma status, the adjusted hazard ratio was 1.08 (95% CI 0.62–1.90; *p* = 0.776), still showing no significant impact of fixation type on union time. After adjusting for confounders, the adjusted survival curve (Fig. 1b) reinforces this finding, with overlapping survival probabilities between plate and IMN groups.

**Table 3:** Hazard ratio for time to union between plate and IMN fixation

Variable	Unadjusted			Adjusted		
	HR	95% CI	*p*-value	HR	95% CI	*p*-value
Plate	0.96	0.57, 1.62	0.893	1.08	0.62, 1.90	0.776
IMN	Reference	Reference	Reference	Reference	Reference	Reference

## Discussion

This study compared the outcomes of IMN and plate fixation for treating humeral shaft fractures, revealing high overall union rates with each method. However, distinct differences emerged in postoperative complications and functional recovery between the two groups. Although the non-union rate was slightly higher in the plate fixation group, this method offered advantages in shoulder function, with significantly better shoulder ROM and reduced incidence of shoulder pain compared to IMN. In contrast, IMN was associated with a lower reoperation rate and higher neuropathy resolution post-surgery. These findings align with the existing literature, indicating that DCP fixation can be advantageous in functional recovery.

Previous studies have reported mixed outcomes when comparing IMN and DCP for humeral shaft fractures. McCormack et al. observed that although both methods achieved similar union rates, IMN was associated with higher functional complications, including shoulder impingement and reoperations [18]. Similarly, Zhao et al. highlighted that, despite its minimally invasive approach, IMN often resulted in more shoulder complications and required more frequent hardware removal than DCP [14]. Our findings are consistent with these studies, demonstrating a higher incidence of postoperative functional complications in the IMN group, while DCP provided superior outcomes regarding shoulder ROM and pain.

In our study, the time to union was comparable between the IMN and plate fixation groups, with no significant differences, as confirmed by the Cox proportional hazards model. The unadjusted and adjusted analyses (accounting for age, gender, and polytrauma status) showed similar union times between the groups, and most patients eventually achieved union. This finding aligns with Esmailiejah et al., who reported comparable union rates between IMN and DCP, though with a tendency toward a longer time to union in the IMN group [19]. Conversely, Amer et al. found a shorter time to union in the IMN group, highlighting variability in outcomes across studies [20].

Infection and nonunion were not observed in either group in our study. These findings are consistent with prior research, such as the work of Chapman et al., who reported similarly low rates if infection and nonunion for both IMN and DCP [21]. Despite the technical challenges associated with DCP, such as the need for extensive dissection, our findings reinforce the reliability of this technique in achieving successful union outcomes without increased risk of infection.

Given the higher rates of functional complication associated with IMN, our study suggests that DCP might be the preferred method for treating humeral shaft fractures, particularly in patients for whom preserving shoulder function is essential. However, IMN remains a viable option in specific cases where the patient's anatomy or fracture characteristics make DCP less practical.

This study has some limitations that should be considered when interpreting the results. The retrospective design primarily leads to challenges with missing data and a limited set of available variables and the choice of implant was based on clinical judgment and fracture configuration. Furthermore, the small sample size may reduce the statistical power to detect significant differences between techniques. The lack of long-term follow-up data also prevents a comprehensive evaluation of the long-term outcomes associated with each approach. Future research should include larger sample sizes and multicenter randomized controlled trials with extended follow-up periods to validate our findings.

## Conclusion

In conclusion, while IMN and DCP effectively achieve fracture union in humeral shaft fractures, DCP is associated with better functional outcomes. However, it was associated with higher rates of re-operation. These findings support the continued use of DCP as a primary treatment method for humeral shaft fractures, with IMN reserved for specific clinical situations where it may offer distinct advantages.

## Data Availability

No datasets were generated or analysed during the current study.
